# Rapamycin Inhibits Cardiac Hypertrophy by Promoting Autophagy via the MEK/ERK/Beclin-1 Pathway

**DOI:** 10.3389/fphys.2016.00104

**Published:** 2016-03-18

**Authors:** Jun Gu, Wei Hu, Zhi-Ping Song, Yue-Guang Chen, Da-Dong Zhang, Chang-Qian Wang

**Affiliations:** ^1^Department of Cardiology, Shanghai Ninth People's Hospital, Shanghai Jiaotong University School of MedicineShanghai, China; ^2^Department of Cardiology, Shanghai Minhang Hospital, Fudan UniversityShanghai, China

**Keywords:** rapamycin, cardiac hypertrophy, autophagy, MEK/ERK signaling, aortic banding

## Abstract

Rapamycin, also known as sirolimus, is an antifungal agent and immunosuppressant drug used to prevent organ rejection in transplantation. However, little is known about the role of rapamycin in cardiac hypertrophy and the signaling pathways involved. Here, the effect of rapamycin was examined using phenylephrine (PE) induced cardiomyocyte hypertrophy *in vitro* and in a rat model of aortic banding (AB) - induced hypertrophy *in vivo*. Inhibition of MEK/ERK signaling reversed the effect of rapamycin on the up-regulation of LC3-II, Beclin-1 and Noxa, and the down-regulation of Mcl-1 and p62. Silencing of Noxa or Beclin-1 suppressed rapamycin-induced autophagy, and co-immunoprecipitation experiments showed that Noxa abolishes the inhibitory effect of Mcl-1 on Beclin-1, promoting autophagy. *In vivo* experiments showed that rapamycin decreased AB-induced cardiac hypertrophy in a MEK/ERK dependent manner. Taken together, our results indicate that rapamycin attenuates cardiac hypertrophy by promoting autophagy through a mechanism involving the modulation of Noxa and Beclin-1 expression by the MEK/ERK signaling pathway.

## Introduction

Cardiac hypertrophy is an enlargement of the heart in response to different pathological stimuli such as hypertension, myocardial ischemia, and endocrine disorders (Frey and Olson, 2003). Although it is an adaptive response to an increased workload mediated by an increase in muscle mass to maintain cardiac output, it generally progresses to cardiac remodeling and heart failure and it is considered a risk factor for cardiovascular disease and mortality (Lips et al., 2003; Li et al., 2015). The mechanisms underlying cardiac hypertrophy have been investigated to improve the design of therapeutic strategies for the treatment of heart failure, and recent studies have suggested a link between autophagy dysregulation and cardiac hypertrophy (Li et al., 2015).

Autophagy, a catabolic process by which cytosolic proteins are degraded in the lysosome, plays important roles in cellular homeostasis, development, and defense mechanisms (Wang et al., 2009). Autophagy plays a dual role as a pro- and anti-survival process, and dysregulation of autophagy is associated with several diseases including cancer, neurodegeneration, and cardiomyopathy (Maiuri et al., 2007; Levine and Kroemer, 2008). Although autophagy is increased in heart failure associated with dilated cardiomyopathy, ischemic heart disease, and valvular disease, whether autophagy causes heart failure or is activated in response to heart failure remains unclear (Hein et al., 2003; Li et al., 2014).

Among various pathways known to regulate autophagy in mammalian cells, the best understood are the class I phosphatidylinositol 3-kinase (PI3K)/Akt/mammalian target of rapamycin (mTOR)/p70 ribosomal protein S6 kinase and the Ras/Raf-1/mitogen-activated protein kinase ½(MEK1/2)/ extracellular signal-regulated kinase ½(ERK1/2) signaling pathways (Shinojima et al., 2007). The Akt/mTOR pathway is a negative regulator of autophagy, whereas the ERK1/2 pathway positively regulates it. Both class I and class III PI3Ks are involved in the regulation of autophagy, with class I PI3K playing an inhibitory role, whereas the class III PI3K complex, which includes Beclin-1, promotes autophagy (Petiot et al., 2000). Beclin-1, which was first identified as an interacting partner of the antiapoptotic protein Bcl-2, is an autophagy related protein (its yeast homolog is Atg6) that plays a role in the formation of autophagosomes, which are double-membrane-bound vacuoles that engulf cellular material targeted for degradation (Liang et al., 1998; Kihara et al., 2001; Levine and Kroemer, 2008). Autophagy related genes (Atg) are essential to the process of autophagy and approximately 30 Atg proteins have been characterized in yeast and 10 in humans(Mizushima, 2007). Beclin1/Atg6 and microtubule-associated protein 1 light chain 3 (LC3)/Atg8 are among the Atg proteins necessary for autophagosome formation. During the formation of autophagosomes, cytosolic LC3-I is conjugated to phosphatidylethanolamine to generate LC3-II, which is recruited to the autophagosomal membrane and degraded after the fusion of autophagosomes to lysosomes (Tanida et al., 2008); therefore, the LC3-II/LC3-I ratio is a measure of autophagic activity. Oncogenic H-Ras promotes autophagy by up-regulating Beclin-1 and the Bcl-2 homology-3 (BH3)-only protein Noxa, a pro-apoptotic member of the Bcl-2 family (Elgendy et al., 2011). Binding of Mcl-1 to Beclin-1 interferes with its association with PIK3C3 and inhibits its autophagy-inducing potential.

Autophagy is induced in eukaryotic cells by starvation, and the serine/threonine kinase mTOR is a key regulator of the balance between cell growth and autophagy (Jung et al., 2010). Rapamycin, a bacterial product and inhibitor of mTOR that is also known as sirolimus, was developed as a therapeutic agent because of its immunosuppressant and anti-tumor properties. Rapamycin binds to mTOR and prevents the phosphorylation of its downstream substrates p70 ribosomal protein S6 kinase (P70S6K), eukaryotic translation initiation factor 4E-binding protein 1 (4E-BP1) and other proteins involved in transcription, translation, and cell cycle control and further affecting cell survival or death (Yang et al., 2015; Zhou et al., 2016). In the present study, we examined the effect of rapamycin on cardiac hypertrophy *in vitro* and *in vivo* and explored the underlying mechanisms.

## Materials and methods

### Reagents

Antibodies were obtained from the following companies: GAPDH, ERK, p-ERK, MEK, p-MEK, beclin-1, ANP, BNP, Noxa, LC3-I, LC3-II, Mcl-1, P62, mTOR, p-mTOR, Akt, p-Akt, P70S6K1, and p-P70S6K1 (Sigma, St. Louis, MO), Rapamycin and phenylephrine (PE) (Abcam, Cambridge, USA). DMEM (high glucose) and fetal bovine serum were from Nego (Shanghai, China). Cell lysis buffer (10 ×) was obtained from Cell Signaling Technology (Massachusetts, USA). The RT-PCR kit was purchased from TOYOBO (Shanghai China). Other reagents included DAPI (Roche, Germany), hematoxylin and eosin (H&E, Toronto Chemicals, Toronto, Canada), and trypsin (Sigma). GFP-LC3 was purchased from Biovector Science Lab, Inc. (Beijing, China). All pairs of real-time PCR primers were synthesized by Shenggong Biotechnology (Shanghai, China). Other chemicals and reagents were of analytical grade.

### Animals and ethical statement

Sprague-Dawley rats (8–10 weeks old) were obtained from the Shanghai SLAC Laboratory Animal Co., Ltd. All animals were treated in accordance with the Guide for the Care and Use of Laboratory Animals, and all experiments were approved and performed following the guidelines of Ruijin Hospital, Minhang District Central Hospital ethics committee of China.

### Cell lines and culture conditions

The H9c2 cell line is a subclone of the original clonal cell line, which is derived from embryonic rat heart tissue and holds many cardiomyocyte characteristics. Cells were cultured in Dulbecco's Modified Eagle's medium, supplemented with 10% fetal bovine serum, 100 U/mL penicillin, and 100 mg/mL streptomycin, at 37°C in a humidified incubator containing 95% air and 5% CO_2_. The media was refreshed every 3 days. Cells cultured to approximately 80% confluence were treated with either rapamycin (10 ìM), PE (50 ìM), or PD98059 (2 μM; dissolved in normal saline) alone or in combination under serum-free conditions.

### Western blot analysis

H9c2 cells treated as indicated were lysed and approximately 10 μg of proteins were separated by SDS-PAGE and immunoblotted using conventional procedures as described previously (Cicardo and Dick, 1985). Briefly, the protein samples extracted from H9c2 cells were subjected to sodium dodecyl sulfate-polyacrylamide gel electrophoresis and transferred to a polyvinylidene difluoride membrane. Membranes were blocked with TBST containing 5% milk and incubated with the different primary antibodies as indicated overnight at 4°C. The membranes were then incubated with horseradish peroxidase conjugated secondary antibodies and visualized using the enhanced chemiluminescence system. Densitometric analysis was performed using Scion Image software (Scion, Frederick, MD, USA).

### Measurement of the surface area of cardiomyocytes

The cardiomyocytes were visualized using a charge-coupled device camera (Olympus, Japan) and analyzed using Image-Pro Plus software. For measurements of the cell surface area, 100 cells (or myocardial tissue sections) from randomly selected fields in each group were measured using Image Pro-Plus 6.0.

### Histological analysis

Hearts were excised, washed with saline solution, and placed in 10% formalin. Hearts were cut transversely close to the apex to visualize the left and right ventricles. Several sections of the heart (4–5 μm thick) were prepared and stained with H&E for histopathology and then visualized by light microscopy.

### Construction and infection

For knockdown of MEK, Beclin-1 and Noxa, three small interfering RNA (siRNA) molecules (siMEK, siBeclin-1 and siNoxa) were synthesized by GenScript.The target sequences were 5′-GCCGACTGC AAATACAAGTTT-3′, 5′-GGAGCCATTTAT TGAAACTTT-3′, and 5′-CCGGAGAAT TGGAGACAAATT-3′, respectively. H9c2 cells were transfected with siRNA using the oligofectamine protocol according to the manufacturer's instructions (Invitrogen). A GFP-LC3 vector was used to infect H9c2 cells to monitor puncta formation by immunofluorescence microscopy using a Fluoview 1000 System (Olympus, Irving, TX).

### RNA isolation and semiquantitative RT–PCR

Total RNA was isolated from various pretreated H9c2 cells using TRIzol according to the manufacturer's instructions (Invitrogen, Carlsbad, CA). Equal amounts of RNA were added to a reverse transcriptase reaction mix (Themo Scientific), with oligo-dT as primer. The resulting templates were subjected to PCR using the following specific primers: GAPDH (sense 5′-CATCTTCTCAAA ATTCGAGTGACAA-3′, antisense 5′-AGTAGACTC CACGACATACTCA-3′); ANP (sense 5′-CGTATACAG TGCGGTGTC-3′, antisense 5′-TCCCAGGGGCCGCGCCCG-3′); BNP (sense 5′-CCAGATGATTCTGCTCCT-3′, antisense 5′-GAATTT CGAAGTCTCTCC-3′); MEK (sense 5′-GGCTTC TTCCTTCTCGCCCT-3′, antisense 5′-CTTCCAGTT GCAGGGCACCT-3′); Beclin-1 (sense 5′-CATTAC TTACCACAGCCC-3′, antisense 5′-CATCTGTCT GGCCAGAC-3′); and Noxa (sense 5′-GAAAGG CGCGTCGGAACG-3′, antisense 5′-TGGGCT TGGGCTTCTTCT-3′).

### Immunoprecipitation

For immunoprecipitation, cells were lysed in IP buffer (20 mM Tris-HCl [pH 7.5], 1 mM EDTA, 1 M KCl, 5 mM MgCl2, 10% v/v glycerol, 1% v/v Triton X-100, 0.05% v/v 2-Mercaptoethanol and protease and phosphatase inhibitors). Approximately 1–4 mg of lysate were pre-cleared with protein G beads for 30 min at 4°C and subsequently incubated with antibody pre-bound to protein G beads for 2–16 h at 4°C. The beads were washed three times with NP40 buffer. The immunoprecipitation beads were mixed with 6 × sample buffer, loaded onto SDS-PAGE gels, and analyzed by immunoblotting as described previously.

### Autophagy assay

Autophagy was determined by detection of the processing of the autophagy marker LC3 and fluorescence microscopic detection of the formation of the autophagosomes in cells transfected with GFP-LC3 as described previously (Wang et al., 2009).

### Animal experiments

Cardiac hypertrophy was induced by pressure overload, which was performed by descending aortic banding (AB). In brief, rats were maintained in the animal service center of our institution. Following anesthesia by intraperitoneal injection of 1.5% pentobarbital (W × 0.06), rats had the left thorax opened at the second intercostal space and a 7-0 silk suture ligature was tied around the descending aorta against a 26 gauge needle. Then, the needle was quickly removed. A similar surgery was performed in the sham-operated mice with the exception of aortic banding. To study the effect of rapamycin, the mice were intraperitoneally injected with 0.5 mg/kg rapamycin or 0.1 mg/kg PD98059 once a day for 8 weeks. The control mice received an equal volume of normal saline. Before sacrifice, the mice were anesthetized with 2% isoflurane inhalant.

### Echocardiography

The mice were administered inhalant anesthetics (1.5–2% of isoflurane inhalant mixed with 1 L/min 100% O_2_) to ensure that the anesthetic depth was appropriate. Transthoracic echocardiograms (Visual Sonics Vevo 2100 with a 30-MHz transducer) were performed by an experienced technologist blinded to the study group.

### Statistical analysis

Data are expressed as means ± standard deviation (SD) from at least three independent experiments and were analyzed using one-way ANOVA and Tukey's *post-hoc* test. In all cases, *P* < 0.05 was considered to be statistically significant.

## Results

### PE induced cardiomyocyte hypertrophy downregulates Beclin-1 and Noxa

**Figure 1:**
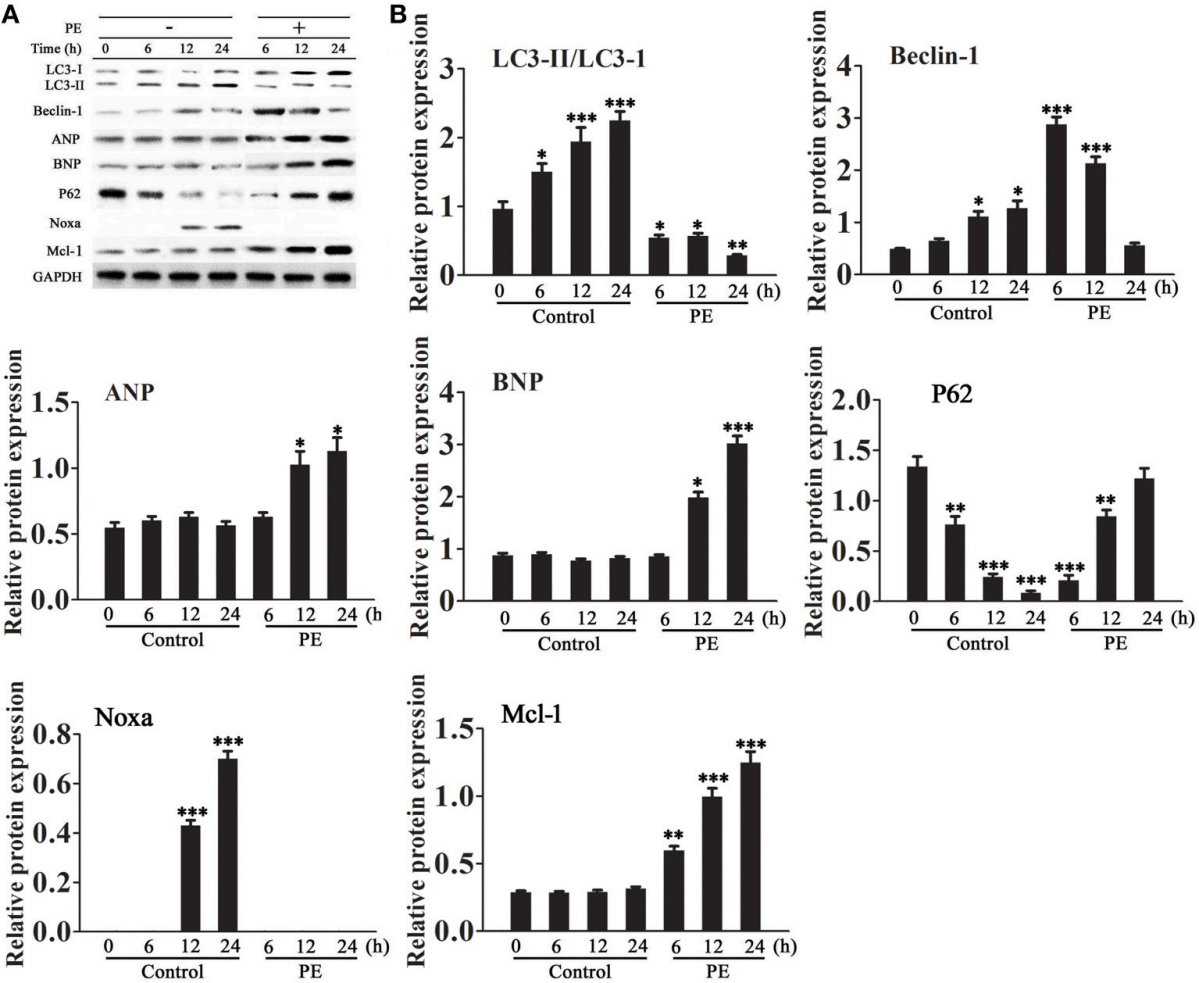
**Effect of phenylephrine on the expression of autophagy and cardiac hypertrophy markers**. Cardiomyocytes were incubated in serum-free medium for 18 h for serum starvation followed by treatment with phenylephrine (PE, 50 ìM) for 6–24 h. **(A,B)** The expression levels of LC3, Beclin-1, Noxa, Mcl-1, ANP, BNP, and P62 were examined by western blotting and quantified by densitometry. GAPDH was used as an internal control. Experiments were repeated four times. ^*^p < 0.05, ^**^p < 0.01, ^***^p < 0.001 vs. Control treated for 1 h.

The expression of autophagy related proteins was examined in a model of PE-induced cardiomyocyte hypertrophy. Treatment of isolated cardiomyocytes with PE is a method used to reproduce the features of pathological hypertrophy, such as increased cell size and sarcomeric re-organization, *in vitro* (Taylor et al., 2000). H9c2 cells were incubated in serum-free medium for 18 h and then treated with or without PE (50 μM) for 6–24 h. Western blot analysis showed that PE decreased the LC3-II/LC3-I ratio, down-regulated Beclin-1 and Noxa, and up-regulated p62, Mcl-1 and the cardiomyocyte hypertrophy markers atrial natriuretic peptide (ANP) and brain natriuretic peptide (BNP) in a time dependent manner (Figures 1A,B).

### Rapamycin inhibits cardiomyocyte hypertrophy by promoting autophagy

**Figure 2:**
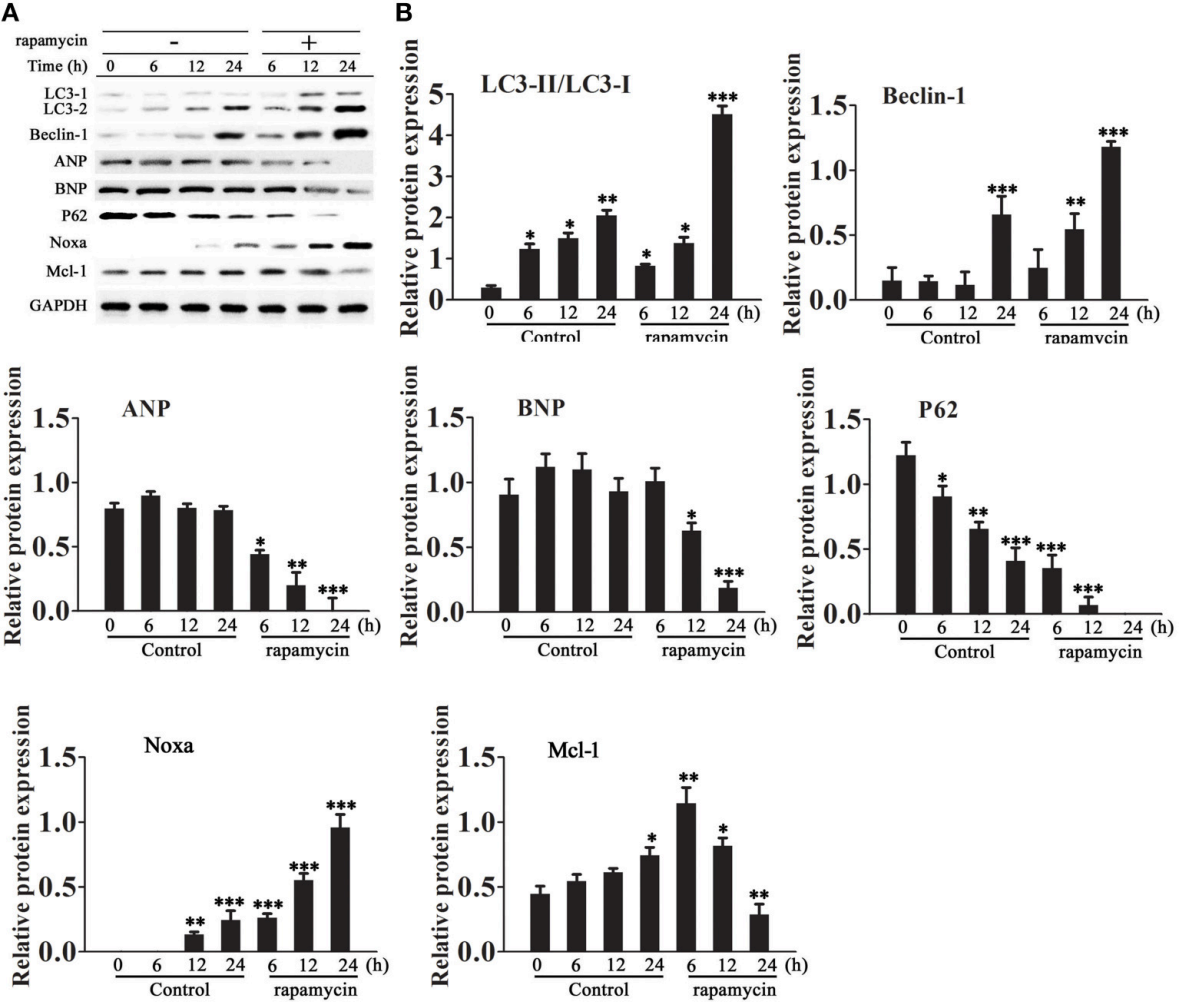
**Effect of rapamycin on the expression of autophagy and cardiac hypertrophy markers**. Cardiomyocytes were incubated in serum-free medium for 18 h followed by treatment with rapamycin (50 ìM) for 6–24 h. **(A,B)** The expression levels of LC3, Beclin-1, Noxa, Mcl-1, ANP, BNP and P62 were examined by western blotting and quantified by densitometry. GAPDH was used as an internal control. Experiments were repeated four times. ^*^p < 0.05, ^**^p < 0.01, ^***^p < 0.001 vs. Control treated for 1 h.

Cardiomyocytes were incubated in serum-free medium for 18 h and then treated with or without rapamycin (50 μM) for 6–24 h. Western blot analysis showed that rapamycin increased the LC3-II/LC3-I ratio, up-regulated Beclin-1 and Noxa, down-regulated p62, and decreased the levels of ANP and BNP and Mcl-1 in a time dependent manner (Figures 2A,B).

### Rapamycin induced myocardial autophagy in a MEK/ERK pathway-dependent manner

**Figure 3:**
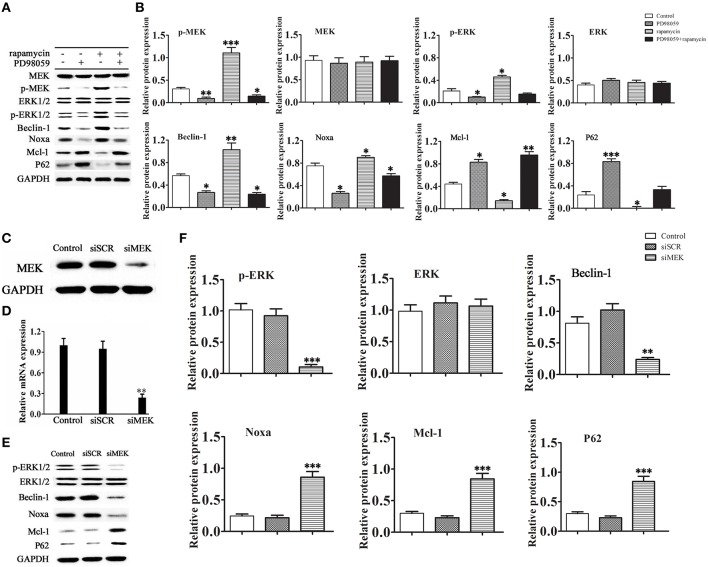
**Rapamycin induces autophagy via activation of the MEK/ERK pathway**. H9C2 cells were treated with rapamycin (10 ìM) for 24 h under conditions of serum depletion, and then treated with or without the MEK inhibitor PD98059 (2 ìM) for 24 h. **(A,B)** Constitutive expression of active MEK or ERK triggered autophagic responses. The expression of the autophagy-related proteins Beclin-1, Noxa, p62 and Mcl-1, and the activation of the MEK/ERK pathway were examined by western blotting and quantified by densitometry. Data represent the mean of three independent experiments. ^*^p < 0.05, ^**^p < 0.01, ^***^p < 0.001 vs. Control. NS means no significant differences vs. Control. (C-F) Western blotting and real-time PCR analyses of the relative protein **(C)** and mRNA **(D)** levels of MEK in cells treated with siRNA against MEK. To determine the expression level of the autophagy-related proteins Beclin-1, Noxa, p62 and Mcl-1, H9C2 cells transduced with or without siRNA against MEK were treated with 10 μM rapamycin in serum-free medium for 2 days. Protein expression was examined by western blotting **(E)** and quantified **(F)** by densitometry. Data represent the mean of three independent experiments. ^**^p < 0.01, ^***^p < 0.001 vs. Control.

To further examine the mechanism by which rapamycin attenuates cardiac hypertrophy, H9C2 cells were treated with or without rapamycin and the MEK inhibitor PD98059, and the activity of the MEK/ERK1/2 pathway and the expression of autophagy markers were assessed by western blotting. The results showed that rapamycin treatment significantly up-regulated the expression of the phosphorylated forms of MEK and ERK1/2, up-regulated Beclin-1 and Noxa, and down-regulated p62 and Mcl-1. These effects were reversed by treatment with PD98059, indicating that rapamycin induces cardiomyocyte autophagy through the activation of the MEK/ERK1/2 pathway (Figures 3A,B). To determine whether the chemical compound affected the results of the experiment, siRNA against MEK was constructed. Figures 3C,D shows the effective down-regulation of MEK at both mRNA and protein levels in response to siRNA treatment. The same experiments were repeated with substitution of PD98059 by siMEK under the induction of rapamycin. The same trend was observed in the results, indicating no differences between the effects of PD98059 and siMEK (Figures 3E,F).

### Rapamycin induced MEK/ERK signaling suppressed the Akt/mTOR/P70S6K1 pathway

**Figure 4:**
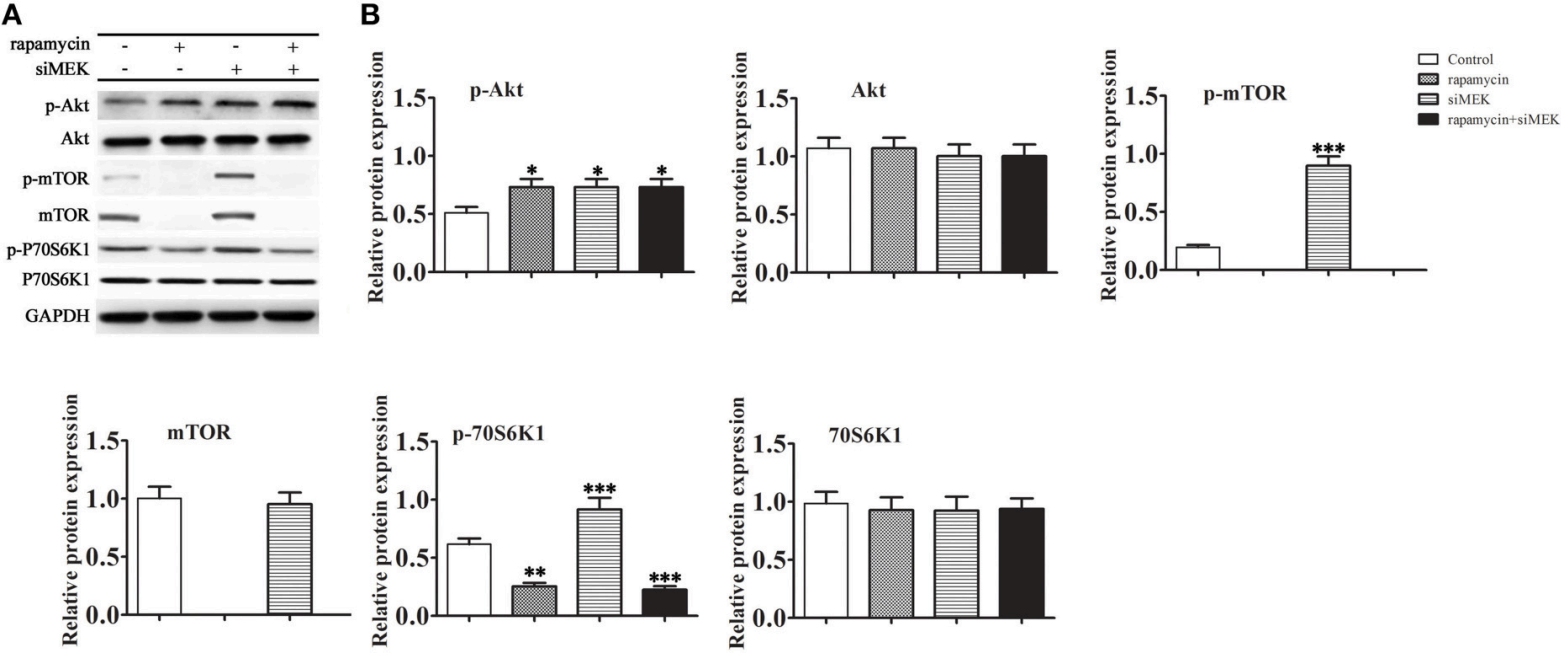
**The effect of rapamycin induced MEK/ERK mediated autophagy to Akt/mTOR/P70S6K1 pathway**. H9C2 cells were treated with rapamycin (10 ìM) for 24 h under conditions of serum depletion, and then treated with or without siRNA against MEK for 24 h. **(A,B)** The expression of the autophagy-related proteins Akt, mTOR, P70S6K1, and the phosphorylation activation of the Akt, mTOR, P70S6K1 were examined by western blotting **(A)** and quantified by densitometry **(B)**. Data represent the mean of three independent experiments. ^*^p < 0.05, ^**^p < 0.01, ^***^p < 0.001 vs. Control.

We next examined whether activation of the MEK/ERK pathway induced by rapamycin had an effect on the Akt/mTOR/P70S6K1 pathway (Figures 4A,B). The results showed that inhibition of MEK by siRNA increased the phosphorylation levels of both Akt and mTOR. However, the total protein level of mTOR decreased markedly in the presence of rapamycin. Under the induction of rapamycin, the phosphorylation level of Akt increased with or without the down-regulation of MEK. These results suggested that the Akt mediated pro-survival signaling pathways mTOR/P70S6K1 were prevented with the inactivation of mTOR by rapamycin.

### Roles of Beclin-1 and Noxa in cardiomyocyte autophagy

**Figure 5:**
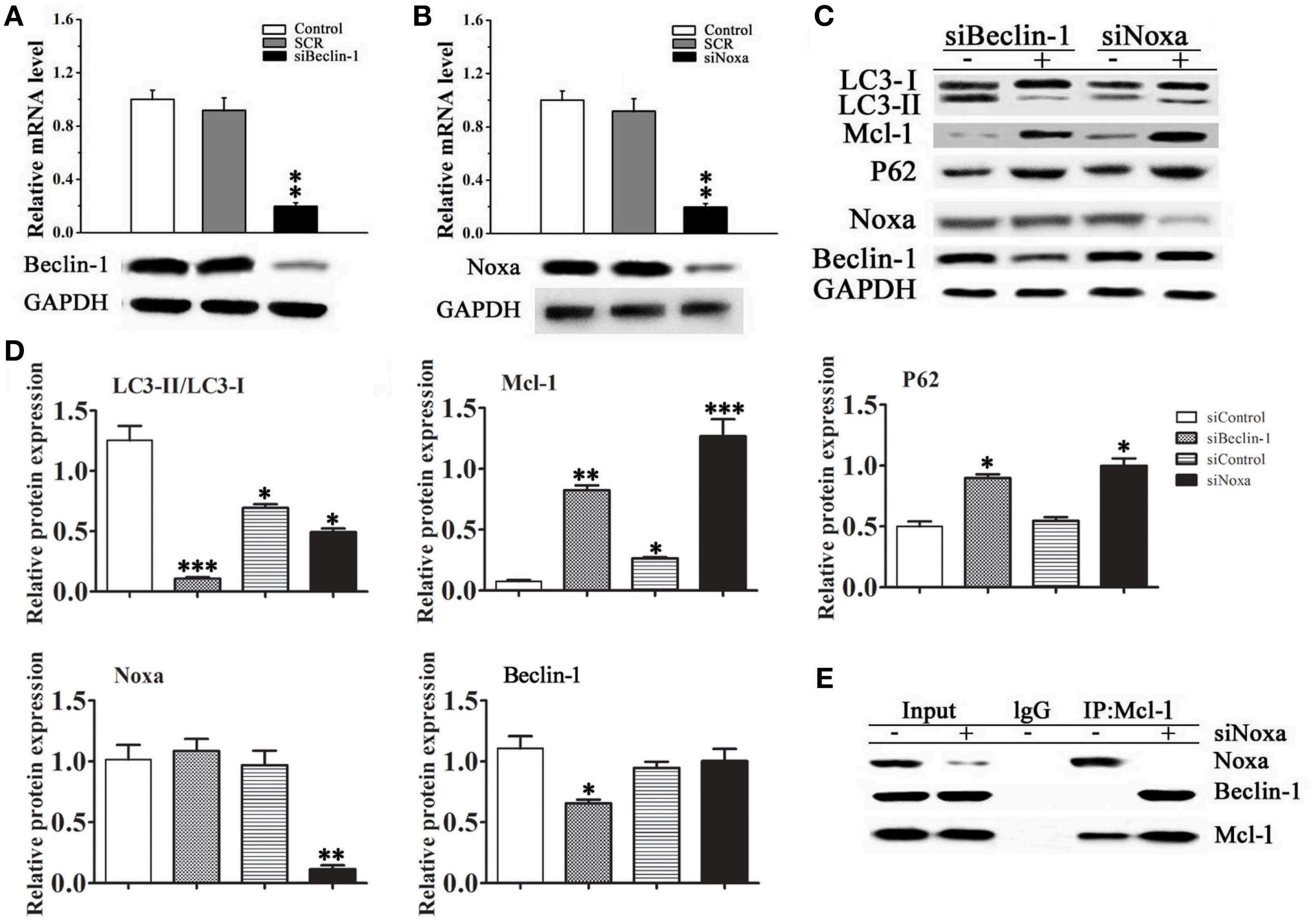
**Noxa plays an important role in Beclin-1 mediated myocardial autophagy**. **(A,B)** The efficiency of siRNA mediated knockdown of Noxa and Beclin-1 in H9C2 cells was examined by RT-PCR. **(C)** The LC3-II/LC3-I ratio and the expression of Mcl-1, p62, Noxa, and Beclin-1 were examined by western blotting in H9C2 cells transduced with or without siRNA against Noxa or Beclin-1 and treated with 10 ìM rapamycin in serum-free media for 2 days. **(D)** Quantification of bands in **(C)**.Data shown are representative of three individual experiments (*n* = 3, mean ± S.E.M.). ^*^p < 0.05, ^**^p < 0.01, ^***^p < 0.001 vs. Control. **(E)** Noxa promotes autophagy by displacing Mcl-1 from Beclin-1. H9C2 cells transfected with control or siRNA Noxa vector were immunoprecipitated with an anti-Mcl-1 antibody or control IgG and immunoblotted against Beclin-1 and Noxa.

The pathway of autophagy in cardiomyocytes was further examined by siRNA mediated knockdown of Beclin-1 and Noxa in H9C2 cells. Figures 5A,B shows the effective down-regulation of Beclin-1 and Noxa in response to siRNA treatment. Silencing of Beclin-1 significantly decreased the LC3-II/LC3-I ratio and up-regulated p62 and Mcl-1 in cells exposed to rapamycin, whereas it had no significant effect on the level of Noxa (Figures 5C,D). Silencing of Noxa decreased the LC3-II/LC3-I ratio, significantly up-regulated p62 and Mcl-1, but had no significant effect on the level of Beclin-1. To understand the roles of Beclin-1 and Noxa on cardiomyocyte autophagy, H9C2 cells transfected with control siRNA or siRNA against Noxa were immunoprecipitated with an anti-Mcl-1 antibody. The results showed that Mcl-1 co-immunoprecipitated with Beclin-1 only when Noxa was silenced by siRNA, suggesting that Noxa promotes autophagy by interfering with the Mcl-1/Beclin-1 interaction (Figure 5E).

### Rapamycin inhibits cardiomyocyte hypertrophy by promoting autophagy via the MEK/ERK1/2 pathway

**Figure 6:**
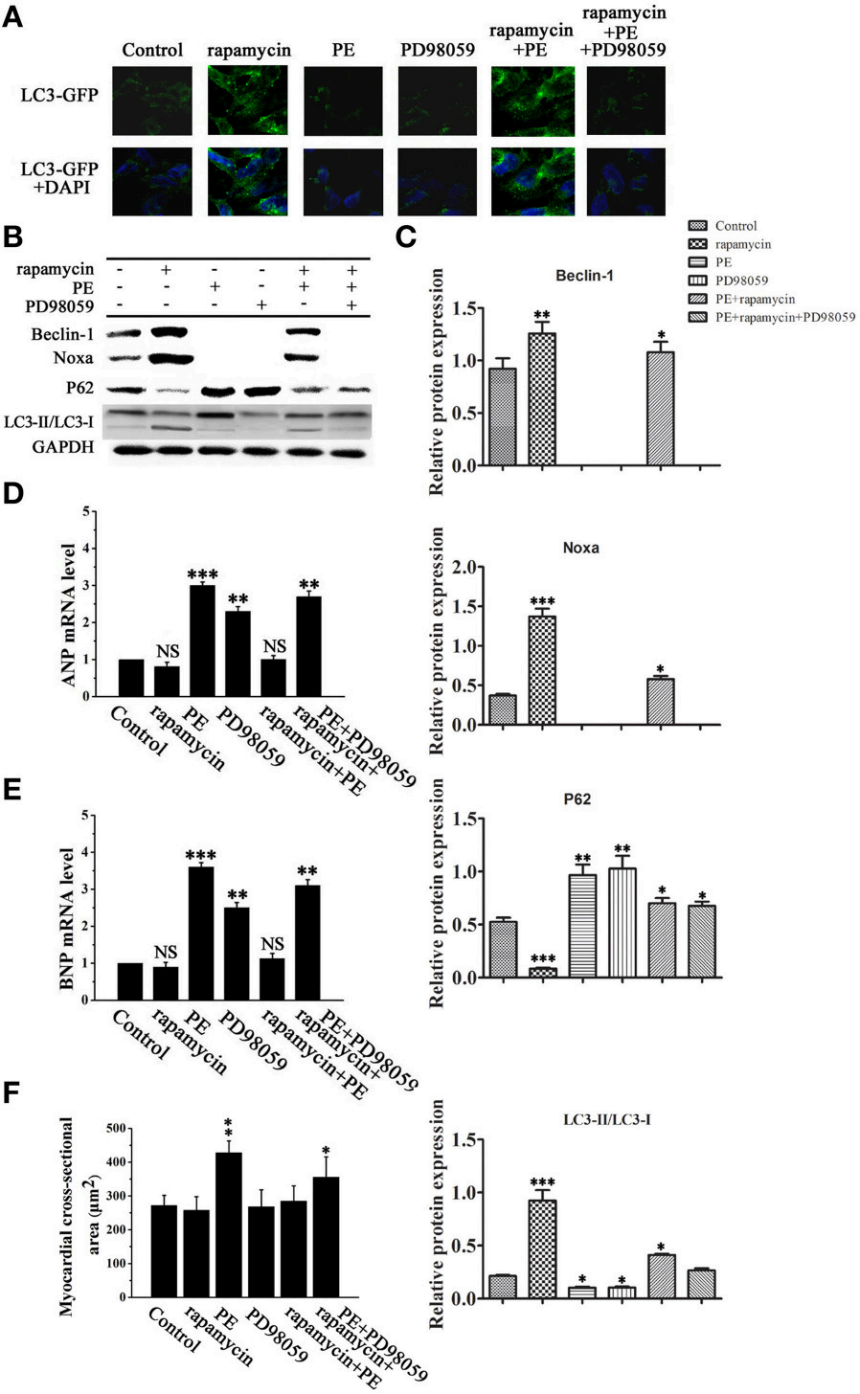
**Rapamycin induced myocardial autophagy protects against cardiomyocyte hypertrophy**. H9C2 cells were treated with rapamycin (10 ìM), PE (50 ìM) or the MEK inhibitor PD98059 (2 μM) and cultured in serum-free media for 2 days. **(A)** Representative fluorescence microscopy images of GFP-LC3 transfected cells treated as indicated**. (B)** Western blot analysis of the levels of LC3-I, LC3-II, p62, Noxa, Mcl-1 and Beclin-1. **(C)** Data shown are representative of three individual experiments. ^*^p < 0.05 vs. Control. **(D,E)** The mRNA expression levels of ANP **(D)** and BNP **(E)** in cardiomyocytes treated as indicated were determined by RT-PCR. ^*^p < 0.05, ^**^p < 0.01, ^***^p < 0.001 vs. Control. NS means no significant differences vs. Control. **(F)** Measurement of myocardial cross-sectional areas in each group. ^*^p < 0.05, ^**^p < 0.01, ^***^p < 0.001 vs. Control.

Figure 6A shows representative images of LC3-GFP immunofluorescent staining of H9C2 cells treated with rapamycin, PE and PD98059. Rapamycin treatment resulted in a perinuclear punctate pattern of LC3-GFP immunofluorescence characteristic of autophagosome formation, which changed to a diffuse pattern in response to PE. Rapamycin reversed the PE-induced inhibition of autophagy, as illustrated by the perinuclear punctate fluorescence, and this effect was suppressed by MEK inhibition, indicating that rapamycin promotes autophagy via the MEK/ERK1/2 signaling pathway in cardyomyocytes under conditions of hypertrophy (Figure 6A). Western blot analysis confirmed these results by showing that PD98059 reversed the rapamycin induced up-regulation of Beclin-1 and Noxa and LC3-II/LC3-I, whereas it significant up-regulated the the levels of p62 (Figures 6B,C). Assessment of the mRNA levels of ANP and BNP by RT-PCR showed that rapamycin suppressed the PE-induced up-regulation of ANP and BNP, and this effect was reversed by PD98059, indicating that rapamycin inhibits cardiomyocyte hypertrophy via the MEK/ERK1/2 pathway (Figures 6D,E). Myocardial cross-sectional area measurements showed that PE treatment increased myocardial size, and blocking rapamycin-induced MEK/ERK1/2 pathway by PD98059 decreased the reduction of myocardial size (Figure 6F). Taken together, these results suggest that rapamycin inhibits cardiac hypertrophy by inducing autophagy via the MEK/ERK1/2 pathway.

### Rapamycin-induced myocardial autophagy protects against cardiac hypertrophy

**Figure 7:**
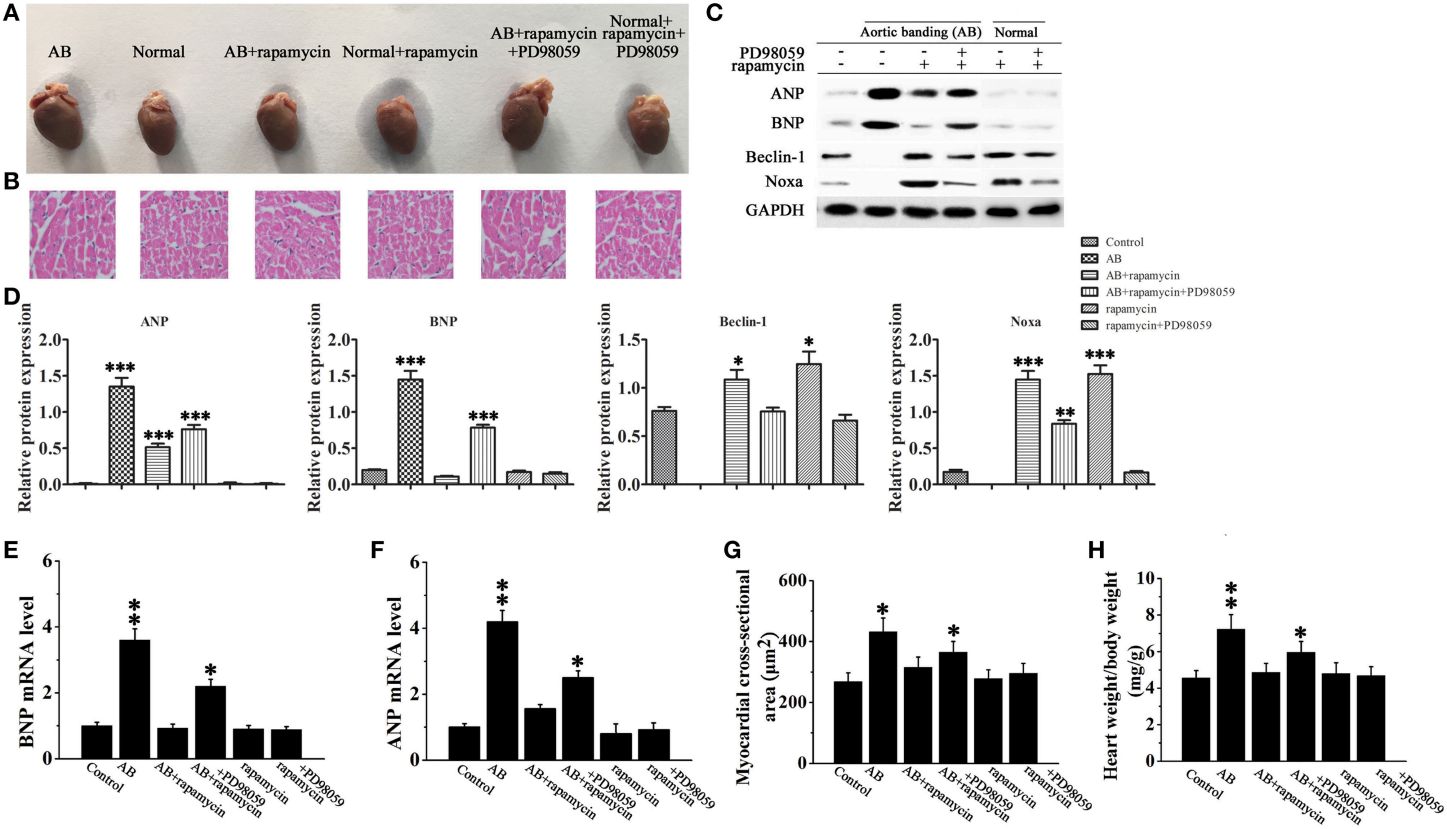
**Rapamycin inhibited cardiac hypertrophy *in vivo***. **(A,B)** Whole hearts **(A)** and H&E staining **(B)** of normal and aortic banding (AB) mice at 8 weeks post-surgery in the presence or absence of rapamycin and/or PD98059. (**C,D**) Western blot analysis of the hypertrophy and autophagy markers ANP, BNP, Beclin-1 and Noxa. ^*^p < 0.05, ^**^p < 0.01, ^***^p < 0.001 vs. Control. Experiments were repeated three times, *n* = 3. **(E,F)** The mRNA expression levels of ANP **(F)** and BNP **(E)** in myocardial tissues in each group were measued by Rt-PCR. ^*^p < 0.05, ^**^p < 0.01 vs. Control. **(G,H)** Measurements of myocardial cross-sectional area and heart weight/body weight ratio in the response to the indicated treatments. *n* = 8. ^*^p < 0.05, ^**^p < 0.01 vs. Control.

To verify our results *in vivo*, cardiac hypertrophy was induced by AB in rats, followed by treatment with or without rapamycin and PD98059. Hearts were excised and tissues were processed for western blotting and H&E staining. Figures 7A,B, which shows representative hearts and H&E stained sections, indicates that the effect of rapamycin on attenuating cardiac hypertrophy is reversed in the presence of PD98059, suggesting that rapamycin attenuates cardiac hypertrophy via a MEK dependent mechanism. Rapamycin treatment down-regulated ANP and BNP, and up-regulated the autophagy markers Beclin-1 and Noxa in the hearts of AB rats, and these effects were reversed by MEK inhibition (Figures 7C,D). To confirm that the effect of PD98059 on suppressing the rapamycin-induced inhibition of hypertrophy is mediated by autophagy and not by a direct effect of PD98059, the mRNA levels of ANP and BNP were examined by Rt-PCR (Figures 7E,F). The results showed that PD98059 restored ANP and BNP expression down-regulated by rapamycin. Furthermore, myocardial cross-sectional area and heart weight measurements showed that PD98059 inhibited the attenuation of cardiac hypertrophy induced by rapamycin (Figures 7G,H). Moreover, echocardiography showed that rapamycin can effectively reduce LVEDd and LVEDs compared with the AB group, suggesting that rapamycin induced autophagy can attenuate cardiac dilation. The mice with pressure overload subjected to rapamycin treatment demonstrated restored cardiac function in terms of alleviated EF (47.8 vs. 35.62%). In addition, echocardiography revealed that rapamycin treatment may relieve left ventricle thickness compared with AB group and have no significant different compared with no-treatment group (Table 1). Accordingly, these findings demonstrate that rapamycin attenuates cardiac hypertrophy and improves cardiac function, which are effects that may be related to the induction of autophagy.

**Table 1:** **Echocardiographic data showed the effects of rapamycin on myocardial autophagy induced by AB**.

AB	-	-	-	+	+	+
rapamycin	-	+	+	-	+	+
PD98059	-	-	+	-	-	+
Number	*n* = 8	*n* = 8	*n* = 8	*n* = 8	*n* = 8	*n* = 8
LVEDd (mm)	3.69±0.12	3.72±0.24	3.56±0.18	5.12±0.13^**^	4.16±0.21^*^^$^	5.02±0.62^**^
LVEDs (mm)	2.64±0.34	2.58±0.26	2.75±0.12	4.6±0.42^**^	2.92±0.38^$$^	3.84±0.27^*^
LVPWd (mm)	0.89±0.08	0.91±0.14	0.87±0.08	1.23±0.11^*^	0.95±0.45^$^	1.18±0.02^*^
IVSTd (mm)	0.87±0.05	0.88±0.14	0.85±0.05	1.12±0.31^*^	0.96±0.06^$^	1.09±0.04^*^
EF%	53.6±4.8	50.9±6.3	54.2±4.9	35.62±2.5^**^	47.8±3.9^$$^	39.24±3.1^**^

*AB, LVEDd, left ventricular end-diastolic dimension (diastolic); LVEDs, left ventricular end-systolic dimension (systolic); LVPWd, left ventricular posterior wall (diastolic); IVSTd, interventricular septum thickness (diastolic); EF, ejection fraction. Data was represented as mean ± SEM, n = 8*.

* *P < 0.05*,

** *P < 0.01 vs. no-treatment groups*.

$ *P < 0.05*,

$$ *P < 0.01 vs. AB group*.

Taken together, these results suggest that the protective effect of rapamycin on cardiac hypertrophy is mediated by the induction of autophagy via a mechanism involving the MEK/ERK1/2 signaling pathway.

**Figure 8:**
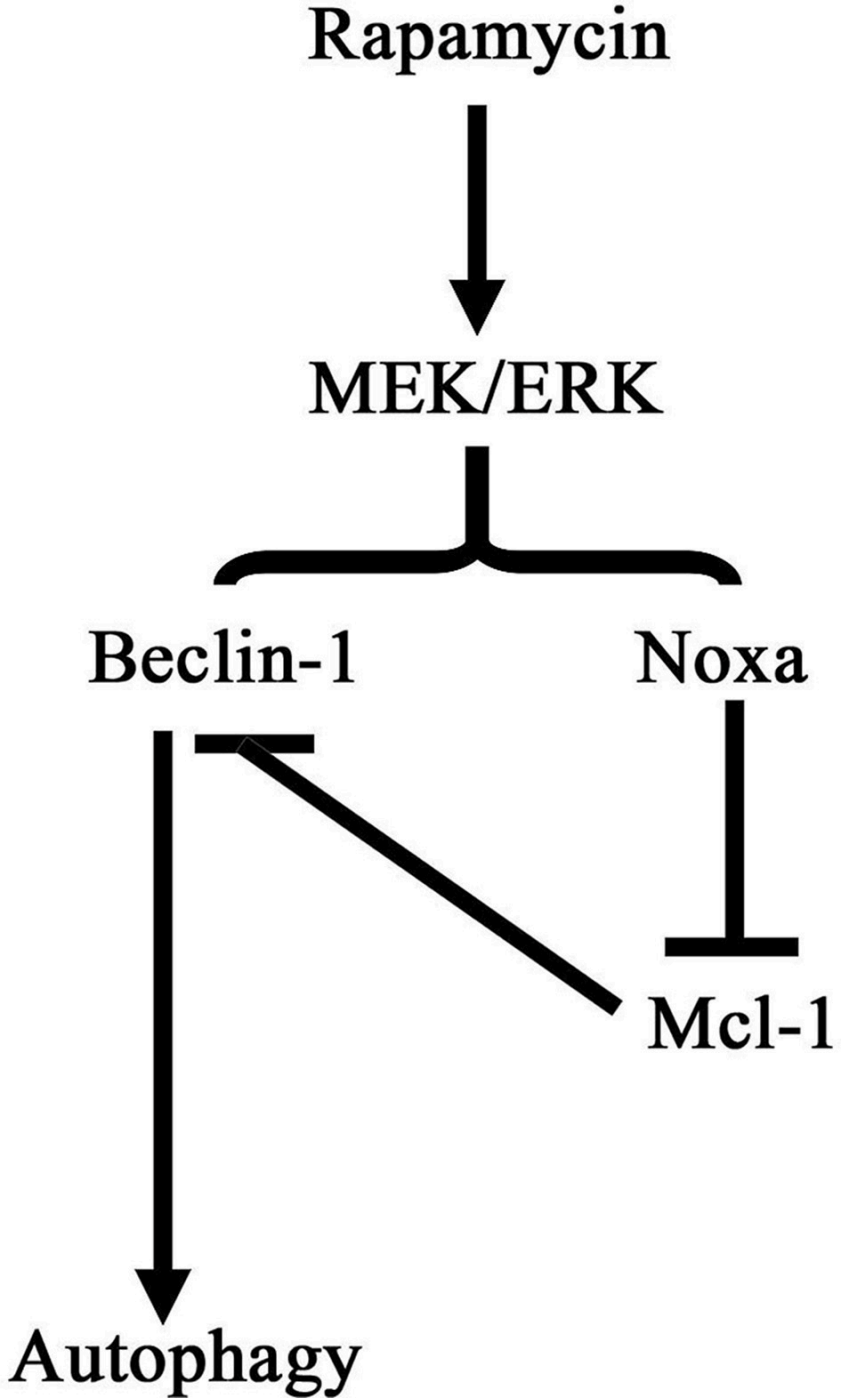
**Schematic illustration of the pathway of autophagy induction by rapamycin in cardiomyocytes**. Rapamycin treatment activates the MEK/ERK signaling pathway, promoting the expression of Beclin-1 and Noxa. Noxa suppressed the inhibitory effect of Mcl-1 on Beclin-1 resulting in the induction of autophagy.

Figure 8 shows a schematic illustration depicting a proposed pathway underlying the effect of rapamycin in our model of cardiac hypertrophy. Rapamycin up-regulates Beclin-1 and Noxa by activating the MEK/ERK pathway leading to the induction of autophagy.

## Discussion

Autophagy plays an important role in the maintenance of cardiac function by removing damaged proteins and subcellular organelles and protecting myocytes under conditions of stress. However, the role of autophagy in the diseased heart is not clear, and whether autophagy promotes cardiomyocyte repair or elimination remains to be determined. In the present study, we showed that rapamycin attenuates cardiac hypertrophy by inducing autophagy and elucidated a potential underlying mechanism involving MEK/ERK12 signaling and the modulation of the expression of Noxa and Beclin-1.

Many signaling pathways regulate autophagy and several of these pathways operate through mTOR, an inhibitor of autophagy. We showed that rapamycin, an inhibitor of mTOR, inhibits cardiac hypertrophy, as determined by the down-regulation of ANP and BNP in cardiomyocytes, and promotes autophagy via the up-regulation of Beclin-1 and Noxa and the down-regulation of p62 and Mcl-1, suggesting that rapamycin exerts a protective effect on cardiomyocytes via the induction of autophagy. Rapamycin was previously shown to inhibit cardiac hypertrophy induced by thyroid hormone treatment or AB (Ha et al., 2005; Kuzman et al., 2007), although the role of autophagy was not addressed. The role of the 5′-AMP-activated protein kinase (AMPK)/mTORC1 pathway in the induction of autophagy in cardiac hypertrophy was reported by Li et al., who showed that AMPK stimulates autophagy by blocking the mTOR complex mTORC1 in a pressure overload model of cardiac hypertrophy (Li et al., 2014). However, AMPK induced autophagy was not mediated by the regulation of Beclin-1, which differs from the present results. The protective effects of rapamycin mediated by the induction of autophagy were demonstrated in mouse models of Huntington's disease, in which inhibition of mTOR promoted the degradation of protein aggregates via autophagy (Ravikumar et al., 2002). Autophagy was shown to have a cardioprotective effect in a mouse model of cecal ligation and puncture, where rapamycin treatment alleviated sepsis-induced cardiac dysfunction via the induction of autophagy (Hsieh et al., 2011). Inhibition of mTOR by everolimus prevents left ventricular remodeling and limits infarct size by inducing autophagy (Hsieh et al., 2011). These studies support the present results showing the protective effect of rapamycin on cardiac hypertrophy mediated by autophagy induction.

In the present study, MEK inhibition reversed the effects of rapamycin on the induction of autophagy and attenuation of PE-induced cardiac hypertrophy, suggesting that rapamycin protects against cardiac hypertrophy by inducing ERK dependent autophagy. These results were confirmed in *in vivo* experiments, which showed the attenuation of cardiac hypertrophy by rapamycin in a MEK/ERK signaling dependent manner. ERK1/2 is among many proteins known to be involved in the regulation of autophagy together with mTOR, AMPK, BH3-only proteins such as Noxa and Beclin-1, and the p62 adaptor protein that binds to LC3(Levine and Kroemer, 2008). Activation of the ERK pathway by curcumin induces autophagy and inhibits the growth of malignant glioma cells (Dong et al., 2015). Activation of the MEK/ERK signaling pathway was shown to trigger autophagy by modulating the expression of Beclin-1 through the dissociation of mTOR complexes 1 and/or 2 in a study that suggested that the protective or destructive roles of autophagy depend on the MEK/ERK-regulated level of Beclin-1 expression (Wang et al., 2009). Noxa is up-regulated by MEK/ERK signaling and plays an important role in autophagy regulation. In melanoma cells, Noxa is up-regulated by oncogenic activation of the MEK/ERK pathway in correlation with constitutive activation of autophagy (Liu et al., 2014). Our co-immunoprecipitation results showed that Mcl-1 interacted with Beclin-1 only in the absence of Noxa, suggesting that Noxa expression suppresses the inhibition of Beclin-1 by Mcl-1 and thus promotes autophagy. PE suppressed Noxa expression, and rapamycin up-regulated Noxa via activation of MEK/ERK signaling. These results suggest that autophagy dysregulation in cardiac hypertrophy could be mediated by the down-regulation of Noxa.Further experiments analyzing the effect of Noxa overexpression are necessary to confirm this hypothesis.

In the present study, silencing of Beclin-1 suppressed rapamycin-induced autophagy. Beclin-1 interacts with antiapoptotic Bcl-2 family proteins, and the functional and physical interaction between Beclin-1 and Bcl-2 is considered important in the crosstalk between autophagy and apoptosis (Pattingre et al., 2005; Nishida et al., 2008). Bcl-2 inhibits Beclin-1 dependent autophagy, and ectopic expression of cardiac Bcl-2 inhibits autophagy in the murine heart by interfering with the formation of the Beclin-1/PI3K complex. The interaction between Bcl-2 and Beclin-1 involves the BH3 domain of Beclin-1, and disruption of this interaction increases autophagy (Maiuri et al., 2007). Overexpression of Beclin-1 stimulates autophagy, and this effect is reversed by Bcl-2 overexpression (Pattingre et al., 2005). These studies are among many exploring the importance of the balance between autophagy and apoptosis in disease. Given the present results showing that the cardioprotective effect of rapamycin is mediated by a MEK/ERK/Beclin-1 axis, it would be of interest to examine the role of apoptosis in our model in future studies.

Dysregulation of autophagy has been demonstrated in cardiovascular disease as well as other diseases, and several FDA-approved drugs induce autophagy, suggesting that targeting autophagy is a potential strategy for the treatment of cardiovascular diseases. Induction of hypertrophy suppresses autophagy, and knockdown of autophagic genes such as Atg5 and Atg7 induces cardiomyocyte hypertrophy (Nakai et al., 2007). Furthermore, induction of autophagy by rapamycin suppresses cardiac hypertrophy in mice (Shioi et al., 2003; Mcmullen et al., 2004). On the other hand, the role of autophagy in ischemia-reperfusion (I-R) injury and heart failure is controversial. While ablation of the proautophagic gene Beclin-1 prevents I-R induced cardiomyocyte death in mice (Matsui et al., 2007), other studies showed that enhancing autophagy through Beclin-1 overexpression has a protective effect against I-R injury (Hamacher-Brady et al., 2006). The protective role of autophagy was supported by studies showing that rapamycin treatment had a cardioprotective role in a mouse model of I-R injury (Khan et al., 2006). Therapeutic modulation of autophagy has been used for the treatment of cardiovascular disorders, and certain cardiovascular drugs such as β-receptor agonists and β-receptor blockers modulate autophagy, although their mechanism of action in inducing autophagy needs to be better understood to harness their beneficial effects. Rapamycin-based drugs, which induce autophagy, are used to prevent restenosis after angioplasty; however, these drugs also lead to the inhibition of endothelial cell repair (Grube and Buellesfeld, 2004; Hayashi et al., 2009). Here, we showed that rapamycin had a protective effect against cardiac hypertrophy and elucidated a potential underlying mechanism mediated by the induction of autophagy via MEK/ERK dependent up-regulation of Beclin-1 and Noxa. Our results suggest that the cardioprotective effects of rapamycin-mediated autophagy induction could be used in the treatment of other cardiovascular disorders and thus merit further investigation.

## Author contributions

WH, CW, and JG: conception and design, collection and assembly of data, data analysis and interpretation, and manuscript writing; ZS and YC: perform the experiment, collection and assembly of data and manuscript writing; DZ: collection of data.

### Conflict of interest statement

The authors declare that the research was conducted in the absence of any commercial or financial relationships that could be construed as a potential conflict of interest.
